# Bacterial invasion into the epidermis of rats with sodium lauryl sulphate‐irritated skin increases damage and induces incontinence‐associated dermatitis

**DOI:** 10.1111/iwj.13864

**Published:** 2022-08-02

**Authors:** Sofoklis Koudounas, Takeo Minematsu, Yuko Mugita, Ayano Nakai, Hiromi Tobe, Chihiro Takizawa, Mao Kunimitsu, Gojiro Nakagami, Hiromi Sanada

**Affiliations:** ^1^ Department of Skincare Science, Graduate School of Medicine The University of Tokyo Tokyo Japan; ^2^ Global Nursing Research Center, Graduate School of Medicine The University of Tokyo Tokyo Japan; ^3^ Department of Gerontological Nursing, Wound Care Management, Graduate School of Medicine The University of Tokyo Tokyo Japan

**Keywords:** bacterial infection, histopathology, incontinence‐associated dermatitis, skin barrier function, urinary‐incontinence

## Abstract

Incontinence‐associated dermatitis (IAD) is caused by prolonged exposure to urine/liquid stool. It is a common and often painful skin condition in older incontinent adults because of poor prevention. Patients with urinary infections are at risk of developing IAD, and to guide the development of novel prevention strategies, we aimed to develop an animal model of IAD by urine and bacteria. First, contralateral sites on the dorsal skin of Sprague–Dawley rats were compromised by sodium lauryl sulphate (SLS), simulating frequent cleansing with soap/water. Filter discs were then placed inside ring‐shaped chambers on foam dressings, inoculated with or without *Pseudomonas aeruginosa*, covered with agarose gels immersed in cultured filtrated urine, and secured in place with an occlusive dressing for 3 days. Untreated and SLS‐compromised sites served as controls. The IAD was developed at bacteria‐inoculated sites, characterised by severe IAD‐like redness that persisted for up to 3 days post‐exposure and higher disruption of the skin barrier function compared with non‐inoculated sites. Pathological changes included epidermal thickening, partial skin loss, inflammatory cell infiltration, accumulation of red blood cells, and invasion of bacteria into the epidermis. This novel, clinically relevant IAD rat model can serve for future prevention developments.

## 1 INTRODUCTION

Incontinence‐associated dermatitis (IAD) is an inflammatory condition that develops from the repeated and prolonged exposure of the skin to urine and liquid stool. The IAD is characterised by persistent redness and inflammation at the skin surface, but if left untreated (UN) can lead to oedema, swelling, and blistering.^1^ The IAD is a common problem in older incontinent adults with reported prevalence and incidence rates ranging from 4.3‐45.7% to 3.4‐25%,^2^, ^3^, ^4^, ^5^, ^6^, ^7^ respectively. Patients often experience pain, discomfort, burning and itching, ultimately extending hospital stays, which is associated with increased health care costs^8^, ^9^ and reduced quality of life.^8^ In addition, IAD is strongly associated with the development of pressure injuries, and despite their different aetiologies, IAD and pressure injuries can coexist^5^, ^10^ in both incontinent and bedridden adults, making adequate treatment and quality of care difficult.

Incontinence to liquid stool is particularly damaging to the skin^1^, ^11^ as a result of its rich composition of digestive enzymes (lipases and proteases), which macerate the skin, impair the barrier function and stimulate cytokine release, whereby bacteria penetrate and enhance damage by increasing inflammation.^12^, ^13^, ^14^ On the contrary, exposure to urine causes mild irritation, maceration, and barrier disruption,^15^, ^16^ but these are not enough to cause IAD. However, malodorous urine, strong‐smelling of ammonia, increases the risk of IAD^17^ by promoting bacterial overgrowth,^18^ which can increase skin pH because of the production of ammonia from the hydrolysis of urea in the urine.

Current prevention strategies focus on minimising skin exposure to incontinence using absorbent products, removing irritants by cleansing with soap and water and protecting the skin with barrier products, which are not sufficient.^19^, ^20^ In particular, these approaches have been linked with disruption of skin barrier functionality by increasing transepidermal water loss (TEWL) and by influencing skin microclimate, such as temperature and humidity, leading to increased skin sensitivity and susceptibility to damage.^21^, ^22^ For this reason, it is imperative to develop innovative interventions not only to prevent IAD but also to reduce the incidence of pressure injuries. Previous studies focused on digestive enzymes and bacteria,^12^, ^13^, ^23^ and in this study we aimed to develop an appropriate animal model of IAD that reproduces the irritant response of IAD induced by urine and bacteria. This will enable an in‐depth investigation of the pathophysiology and allow us to examine possible targeted prevention strategies.

## 2 MATERIALS AND METHODS

### 2.1 Reagents and antibodies

2X Luria‐Bertani (LB) broth was prepared in distilled water containing 20 g/L peptones (Nakalai Tesque Inc., Kyoto, Japan), 10 g/L yeast extract (Nakalai Tesque Inc., Kyoto, Japan) and 20 g/L sodium chloride (Fujifilm Wako Pure Chemicals, Osaka, Japan) and diluted to 1X with distilled water or synthetic human urine (s‐urine, pH 6.50). 2X s‐urine was used as the moisture irritant source experienced by patients with urinary incontinence, and prepared as previously described^24^ by adding the following chemicals to constantly stirring distilled water: 4% urea, 1.6% sodium chloride, 0.06% magnesium sulphate, and 0.016% calcium chloride. All chemicals were purchased from Fujifilm Wako Pure Chemicals (Osaka, Japan). The LB medium was autoclaved (20 min at 121°C) and s‐urine was sterile filtered (0.45 μm; Starlab, Bagneux, France) before use. Agarose S was purchased from Nippon Gene (Tokyo, Japan) and 1% agarose gels were prepared in 1 M Tris HCl buffer (pH 8.5). Transparent adhesive (Tegaderm™,3 M, St. Paul, Minnesota) and foam dressings (FoamLite™, Convatec, Skillman, New Jersey), and biopsy punches (8 mm, Kay Industries, Oyana, Japan) were also purchased. Ethanol (absolute) and methanol (absolute) were purchased from Fujifilm Wako Pure Chemicals (Osaka, Japan) and G‐NOX, an alternative solvent to xylene, and the paraffin wax were purchased from GenoStaff Co., Ltd (Tokyo, Japan). Sodium lauryl sulphate (SLS) granules were purchased (Nakalai Tesque, Kyoto, Japan) and prepared in distilled water. Primary antibodies were as follows: goat myeloperoxidase (MPO) polyclonal antibody (sc‐34 159; 1:50 dilution) purchased from Santa Cruz Biotechnology (Santa Cruz, California); mouse monoclonal anti‐major histocompatibility complex (MHC) class II (OX‐6, PA1‐73119, 1:200 dilution) and biotin‐conjugated (SP) polyclonal rabbit anti‐Pseudomonas antibody (PA1‐73119, 1∶300 dilutions) purchased from Thermo Fischer Scientific (Waltham, Massachusetts); Secondary antibodies were purchased as follows: Biotin‐SP (long spacer) AffiniPure Donkey Anti‐Mouse IgG (H + L; 715‐065‐150; dilutions 1:1000) from Jackson ImmunoResearch Laboratories and rat anti‐goat Histofine® Simple Stain MAX PO Universal immunoperoxidase polymer (HI1902) from Nichirei Biosciences (Tokyo, Japan). Peroxidase‐conjugated avidin (Vectastain ABC kit®) was purchased from Vector Laboratories (Funakoshi, Tokyo, Japan) and 3,3′‐diaminobenzadine (DAB) chromogen tablets (10 mg/tablet) were purchased from Fujifilm Wako Pure Chemicals (Osaka, Japan) and DAB solution prepared by dissolving 2 tablets with 10% Tris‐HCl (pH 7.6).

### 2.2 Ethics

The experimental protocols for this study were approved by the Animal Research Committee of the University of Tokyo, Japan (Approval Number 19238), and conducted at the local facility in agreement with national and international guidelines for animal welfare, including the Guide for the Care and Use of Laboratory Animals issued by the National Institutes of Health (NIH). Every effort has been made to minimise the number of animals used and their suffering. Accordingly, we considered five animals per group to be sufficient, and contralateral sites in each animal were tested. All procedures were performed under isoflurane anaesthesia (5% for induction and 2.5% for maintenance). We followed the Animal Research: Reporting In Vivo Experiments (ARRIVE) guidelines in reporting this study.^25^

### 2.3 Animals

A total of 20 male Sprague–Dawley rats (≥6 months old) were purchased from Japan SLC (Shizuoka, Japan) and housed in individual cages in the local animal facility under controlled pathogen‐free conditions, with the temperature of 23°C ± 2°C and humidity of 50% ± 10%, on a 12‐hour light/dark cycle with ad libitum access to food and water. Animals were acclimatised for at least 7 days, and dorsal fur of rats was shaved with an electric shaver, and the remaining hair was completely removed with depilatory cream 2 days before experiments.

### 2.4 Preparation of cultured urine filtrate and bacterial inoculum

A clinical isolate of *Pseudomonas aeruginosa* (PAO1, NITE Biological Research Center 106052) was obtained from the National Institute of Technology and Evaluation, Biological Resource Centre (Chiba, Japan), as it is commonly found in the perineal skin of incontinent patients and can cause urinary tract infection,^26^ and stored at −80°C in LB broth with glycerol (15%). *P. aeruginosa* pre‐cultures were grown in liquid LB overnight at 37°C with continuous shaking for 24 hours. For the preparation of cultured urine filtrate (CUF), 100 μL of pre‐culture were added to 10 mL liquid LB supplemented with s‐urine (1:1) for ~9 hours at 37°C with shaking and filtered (0.45 μm; Starlab, Bagneux, France) to remove bacteria. This was chosen on the basis of results which reported that bacteria show enhanced growth after 8 hours of cultivation.^18^ Similarly, the bacterial inoculum was prepared by overnight (~18 hours) growth of 100 μL pre‐culture in 10 mL of liquid LB with s‐urine at 1:1 ratio at 37°C with shaking. Both CUF solution and inoculum had a pH of 7.25 and 7.37, respectively.

### 2.5 Animal treatment

**FIGURE 1:**
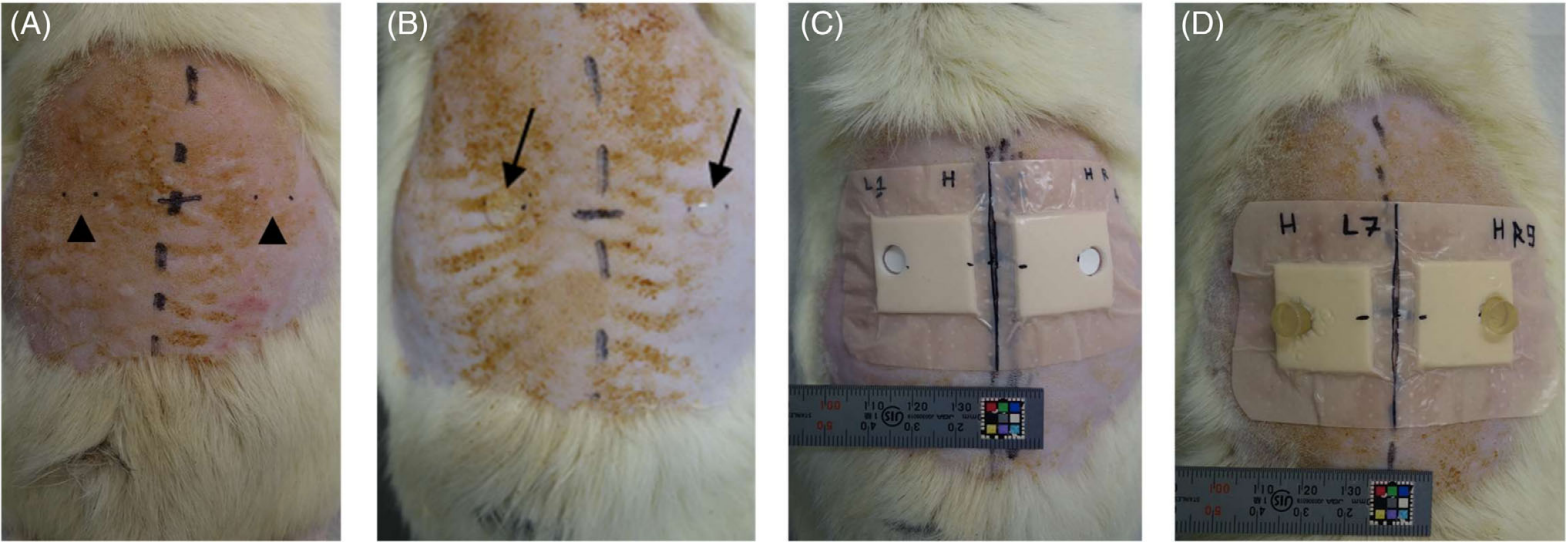
Animal treatment procedure. (A) Contralateral sites marked on dorsal skin, (B) agarose gels immersed in 2% sodium lauryl sulphate were applied under occlusion for 4 hours, (C) 8 mm chambers were punched out of a foam dressing and filter paper discs placed inside, (D) 50 μL bacterial suspension was spotted on filter discs, with non‐inoculated and control sites receiving no treatment, and then agarose gels immersed in cultured urine filtrate were applied onto the filter papers and secured in place with a clear occlusive dressing for 3 days

As the skin of older adults is characterised by reduced barrier functionality and a diminished buffering capacity, which is responsible for the tight regulation of skin pH,^27^, ^28^, ^29^ in order to induce IAD, animal skin was compromised with SLS. Experimental animals were randomly divided into four groups (n = 5 animals/group). The groups were: (1) UN, (2) SLS, (3) SLS + CUF, and (4) SLS + CUF + PAO1 inoculation (CUFB). Briefly, contralateral sites at an equal distance (20 mm) from the midpoint (from the bottom of the shoulder blade to the top of the femur) on the dorsal skin were marked to the right and left, separated by 40 mm (Figure 1A), and the animals in the SLS, CUF and CUFB groups were pre‐treated by applying agarose gels immersed in 2% SLS on marked areas under occlusive conditions for 4 hours (Figure 1B). Subsequently, 8 mm chambers were punched out of foam dressings and applied to tested areas, and filter paper discs (7 mm) were placed inside the chambers (Figure 1C) and inoculated with and without 50 μL bacterial suspension. To ensure long time maceration, a critical factor for IAD development,^13^ 1% agarose gels, after overnight immersion at 4°C in CUF, were applied (Figure 1D) and secured in place with a transparent dressing for 3 days.

### 2.6 Skin assessment

Animal skin was macroscopically examined daily with a digital camera (NEX‐3, Sony, Tokyo, Japan) and the erythema index (EI) was quantified, corresponding to the intensity of skin redness, with Image J 1.53e software (NIH, Bethesda, Maryland).^30^ Skin barrier function was evaluated by quantifying TEWL using the closed chamber VAPO SCAN (AS‐VT100RS, Asch Japan Co. Ltd., Tokyo, Japan) and skin surface pH was measured with a digital glass electrode (Hanna Instruments, HI 981037, Japan). These were measured on day 0, before treatment (normal barrier function values) and after SLS challenge (compromised barrier function values), on day 3 (after treatment), and then every day up to day 7. Three repeat measurements were recorded at the same anatomical location.

### 2.7 Histological analysis

On day 3, tissue samples were harvested from 10 animals and the rats were sacrificed by CO_2_ asphyxiation. To avoid tissue damage from the procedure, a 20 × 20 mm square was marked with the exposed area within and in the middle. Tissues were fixed in 10% neutral‐buffered formalin overnight at room temperature, placed in histological cassettes, immersed in 70% ethanol for 1.5 hours at 4°C, dehydrated through a gradual series of alcohol, embedded in paraffin blocks, and stored at 4°C until staining. From each paraffin block, ~3 μm sections were cut using a sliding microtome (Retoratome REM‐710, Yamato Kohki Industrial, Saitama, Japan), deparaffinised in G‐NOX, rehydrated in ethanol (100%), and stained with haematoxylin and eosin (H & E), and by immunohistochemistry for markers of neutrophil (MPO) and macrophage (major histocompatibility complex Class II; MHC‐II) infiltration, and *Pseudomonas* bacteria as follows. After quenching the endogenous peroxidase activity by incubation in 0.3% H_2_O_2_ in methanol for 30 min, heat‐induced antigen retrieval (MPO and MHC‐II only, not required for PAO1) was performed by autoclave (121°C for 15 min) in citrate buffer (pH 6.0). All sections were then blocked with 1% wt/vol Bovine Serum Albumin (BSA) in phosphate buffered saline (PBS) for 30 min, followed by overnight incubation with primary antibodies at 4°C. The next day, secondary antibodies were applied and incubated for 30 minutes, and immunoreactions (only for MHC‐II and PAO1) were amplified using the Vectastain ABC kit®. All sections were then visualised by DAB solution, which resulted in a dark brown precipitate, and counterstained with haematoxylin. All incubations were performed in a humid chamber at room temperature (unless otherwise stated) and slides were washed with phosphate‐buffered saline between steps. Mounted slides were examined using a digital microscope (BZ‐X710, KEYENCE, Osaka, Japan).

### 2.8 Data analysis

Data are expressed as means ± SD, and graphs overlaid with scatterplots to show the sample distribution were created in GraphPad Prism 8 (GraphPad Software, San Diego, California). Quantification of histology was performed in Image J and all images were scale calibrated. Epidermal thickness was determined in H&E images by measuring the length of 10 straight lines between the skin surface and the basement membrane. Neutrophil and macrophage infiltration was assessed by counting the number of positively stained cells in five 0.1 × 0.1 mm areas per section using the software's cell counter plugin. SPSS version 25 (IBM Corporation, Armonk, New York) was used for statistical analysis, and differences between groups were assessed with one‐way analysis of variance (ANOVA), followed by Tukey's post‐hoc test for pairwise comparison when the ANOVA was significant. Changes from baseline in TEWL and skin pH after exposure to SLS were examined by paired *t*‐tests. *P* value equal to or less than .05 was considered statistically significant. An electronic laboratory notebook was not used.

## 3 RESULTS

### 3.1 Compromised skin barrier by SLS

To confirm skin barrier disruption after SLS exposure, TEWL and skin pH were measured. A significant increase in both parameters compared with baseline values was observed (*P* < .001 in both cases). In particular, the mean values of TEWL and pH increased from 5.27 ± 3.40 to 65.18 ± 16.73 g/h/m^2^ and from 6.78 ± 0.51 to 7.89 ± 0.22, respectively.

### 3.2 Skin appearance and barrier disruption

**FIGURE 2:**
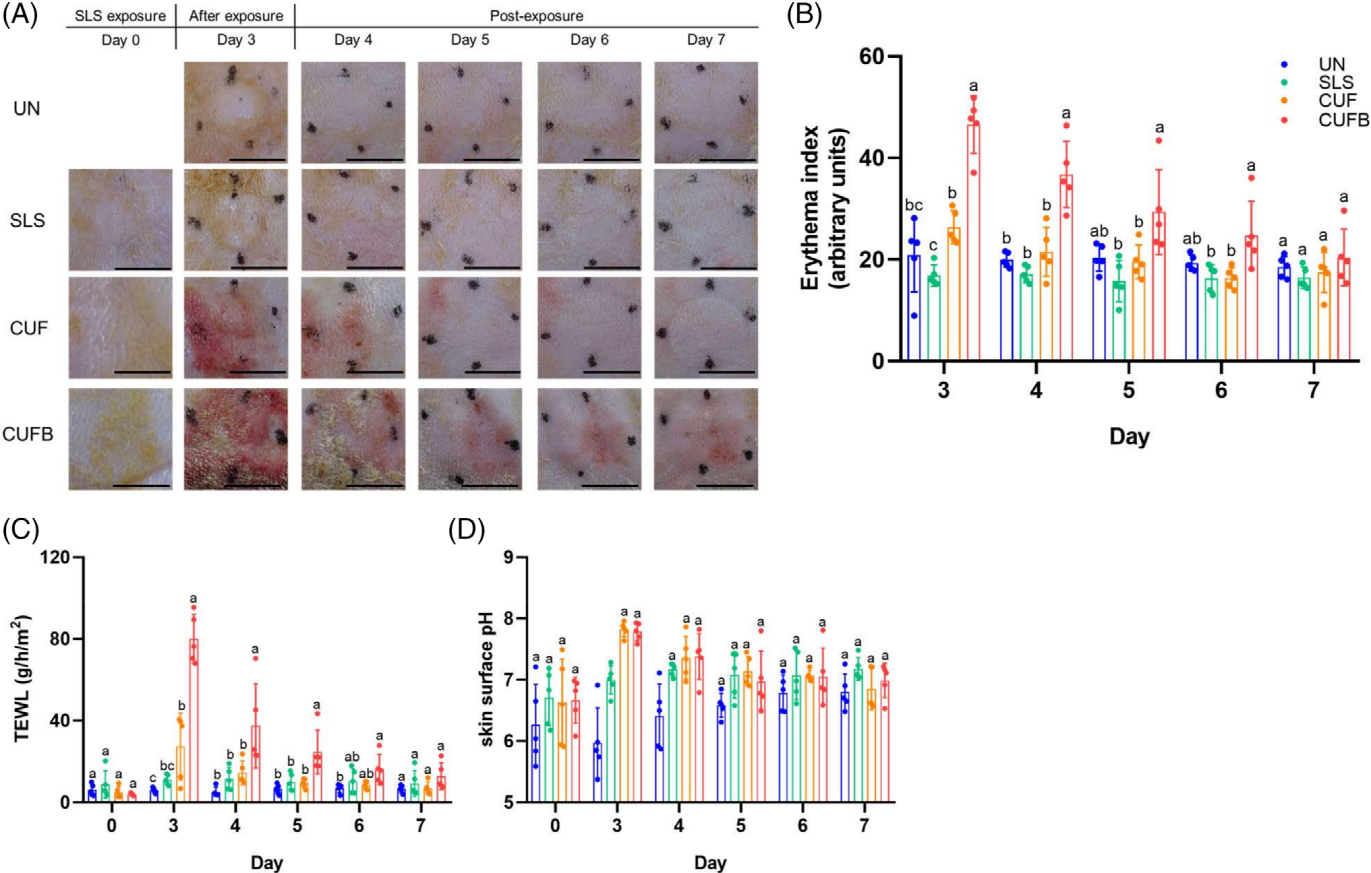
Representative images of skin appearance and quantitative measurements of erythema index (EI), transepidermal water loss (TEWL) and skin pH in control and treated rats. (A) Macroscopic examination. There were no abnormalities in control rats (untreated [UN] and sodium lauryl sulphate [SLS]); temporary mild redness was observed in rats treated with CUF, while rats exposed to the combination of cultured urine filtrate (CUF) and bacteria (CUFB) developed severe redness that was persistent until day 6. (B) Quantification of EI. The EI was significantly increased in CUFB‐treated rats after the exposure period (d3) compared with all other groups. For the CUF group, a significant increase was observed only when compared with SLS (*P* = .036). At the sites treated with CUFB, EI values were significantly higher up to day 6 compared with CUF group (*P* > .001 in all cases for days 3, 4 and 5; *P* > .05 for day 6). (C) TEWL measurement. TEWL was significantly increased in animals treated with both CUF and CUFB. In rats treated with CUF, after exposure, TEWL was only significant in comparison with the UN group (*P* = .021). The highest increases in TEWL were observed in rats treated with CUFB, and these were statistically significant compared with all other groups (*P* < .001 for day 3, *P* > .05 for days 4 and 5). On day 6, TEWL value returns back to baseline levels. (D) Skin pH measurement. CUF and CUFB treatments caused a significant increase on days 3 and 4 compared with control groups (*P* > .001 in all cases), but no significant differences were found between them (*P* = .997). Values with the same letter are not significantly different at *P* ≤ .05

After 3 days, macroscopic examination (Figure 2A) showed no abnormalities or redness in control rats (UN and SLS groups), although signs of dryness were observed in all SLS‐exposed animals. Animals treated with CUF developed mild redness, evident by the increase in EI values (Figure 2B), but the difference was significant only when compared with the SLS group (*P* = .036). The redness in the CUF group was transient and resolved within 24 hours. In contrast, animals that received bacterial inoculation (CUFB group) developed severe redness, which was also reflected by the significant elevation of the EI compared with the other groups (*P* < .001 in all cases) which persisted for up to 4 days post‐exposure. Skin barrier parameters, TEWL (Figure 2C) and skin pH (Figure 2D) were increased in all animals in CUF and CUFB groups. In particular, animals treated with CUF, demonstrated significantly higher TEWL values compared with the UN group (*P* = .021), while the highest TEWL increases were detected at all the inoculated sites, and the differences were statistically significant compared with all other groups (*P* < .001, in all cases). Elevated TEWL values in the CUFB group were maintained until day 5, with TEWL returning to basal levels on days 6 and 7. A rise in skin pH was also observed in the rats of CUF and CUFB groups compared with both control groups (*P* > .001 in all cases). However, there was no significant difference in the degree of skin pH increase between the CUF and CUFB treated animals (*P* = .997). On day 5, there were no significant differences between the groups.

### 3.3 Histopathological findings

**FIGURE 3:**
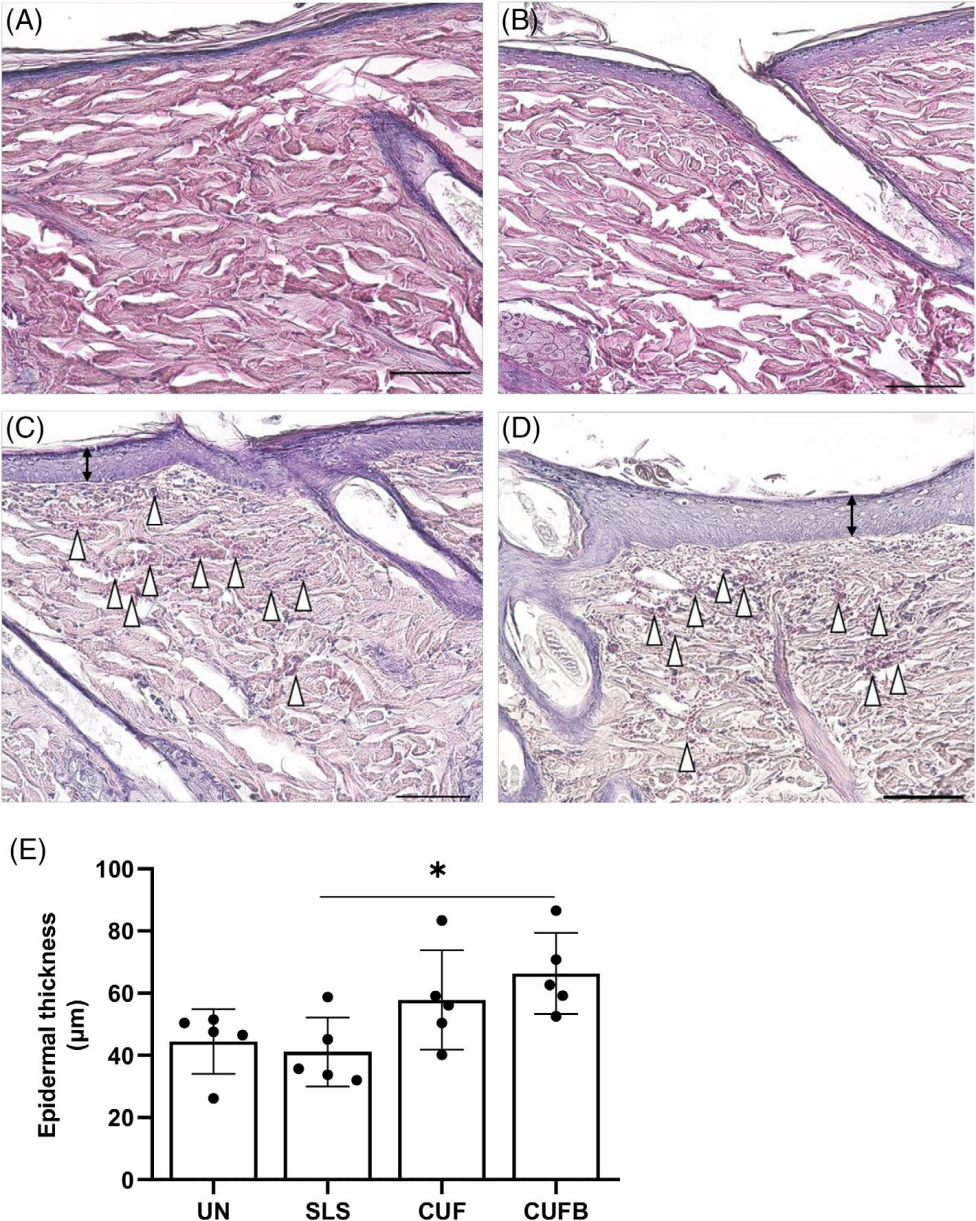
Representative images of haematoxylin and eosin (H&E) staining, and epidermal thickness of skin in control and treated rats (magnification: ×20). Rats in untreated (A) and sodium lauryl sulphate (SLS) (B) groups showed normal skin histology with a visible stratum corneum. Rats treated with cultured urine filtrate (CUF) (C) and CUFB (D) showed a disturbed skin structure characterised by sloughing of the stratum corneum, thickening of the epidermis (black double‐headed arrows), increased granular layer (purple colour), abnormal keratinocyte differentiation, and accumulation of red blood cells in the dermis (white arrowheads). Collagen fibres in the dermis also appear thinner, disorganised, and surrounded by large white areas. Scale bar: 100 μm. (E) Quantification of epidermal thickness. Increased epidermal thickening was observed in CUF and CUFB treated rats compared with control rats. Differences between SLS and CUFB were only significant (*P* = .031)

**FIGURE 4:**
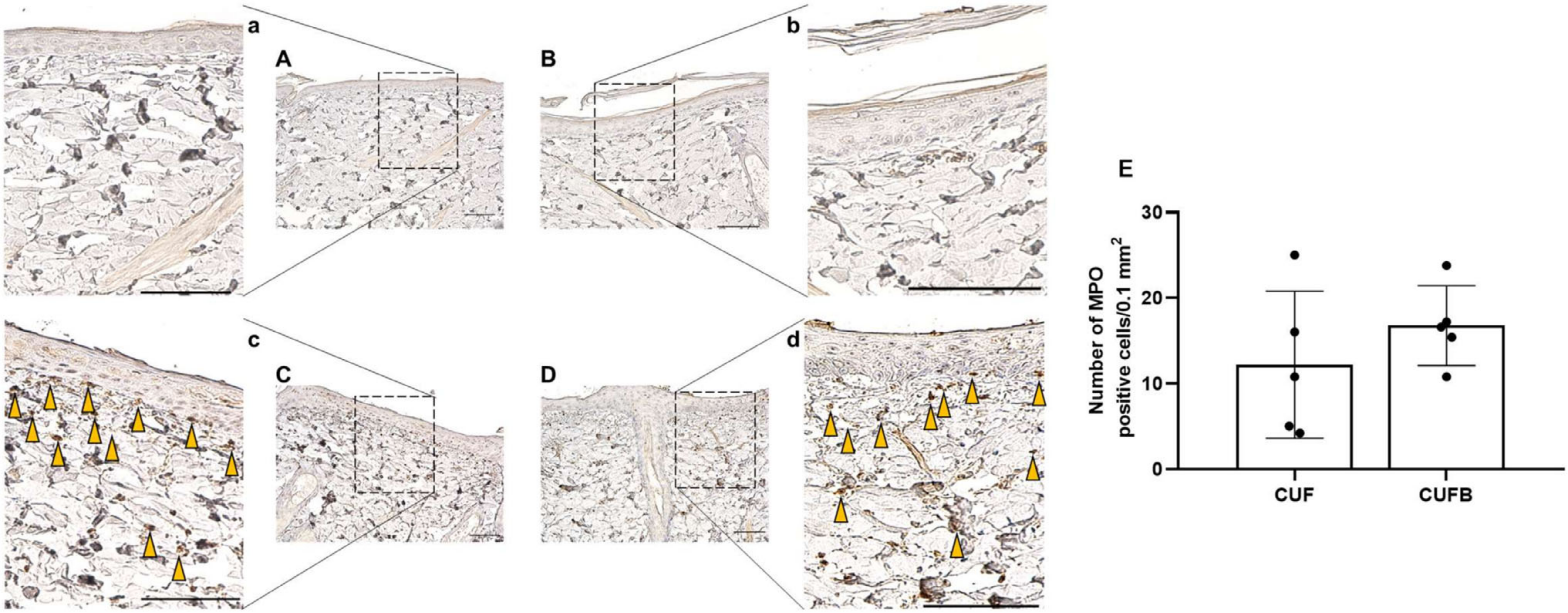
Representative images of immunohistochemistry for the neutrophil marker myeloperoxidase (MPO; magnification: ×20). Brown colour indicates cells with positive expression. No expression of MPO was detected in untreated (A) and sodium lauryl sulphate (B) tissues. Infiltrating inflammatory cells in the dermis of rats treated with cultured urine filtrate (CUF) (C) and CUFB (D) stain positive for MPO (yellow arrows). (a‐d) Magnified images of boxed areas in A‐D. Scale bar = 100 μm. (E) Quantification of average number of MPO positive cells per 0.1 mm^2^ in CUF and CUFB treated rats. No significant differences were found (*P* = .328)

**FIGURE 5:**
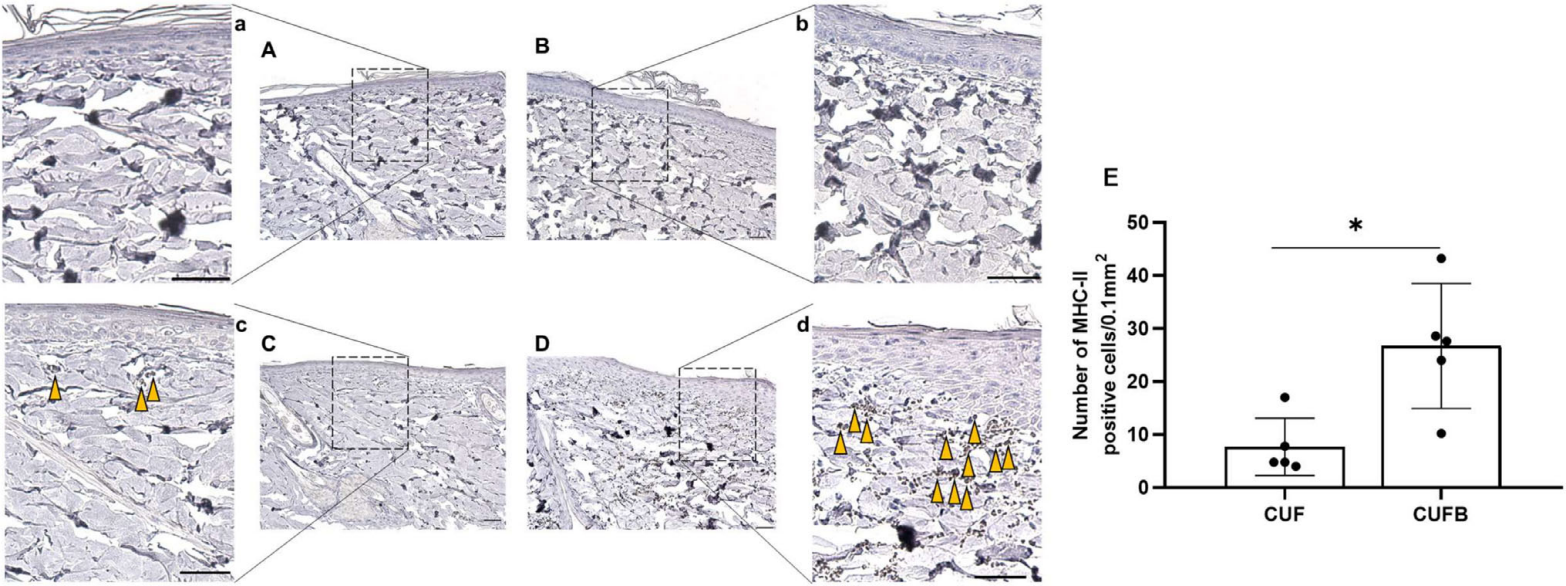
Representative images of Immunohistochemistry staining for the macrophage marker MHC‐II (magnification: ×20). Brown colour indicates cells with positive expression. Untreated (A) and sodium lauryl sulphate (B) tissues showed a negative result with no expression of MHC‐II. MHC‐II expression was detected in the dermis of rats treated with cultured urine filtrate (CUF) (C) and CUFB (D), as shown by the yellow arrows, with larger MHC‐II positively stained areas observed in (D). (a‐d) Magnified images of boxed areas in A‐D. Scale bar = 50 μm. (E) Quantification of average number of myeloperoxidase positive cells per 0.1 mm^2^ in treated rats. Significant differences were observed between the CUF and CUFB groups (*P* = .011)

**FIGURE 6:**
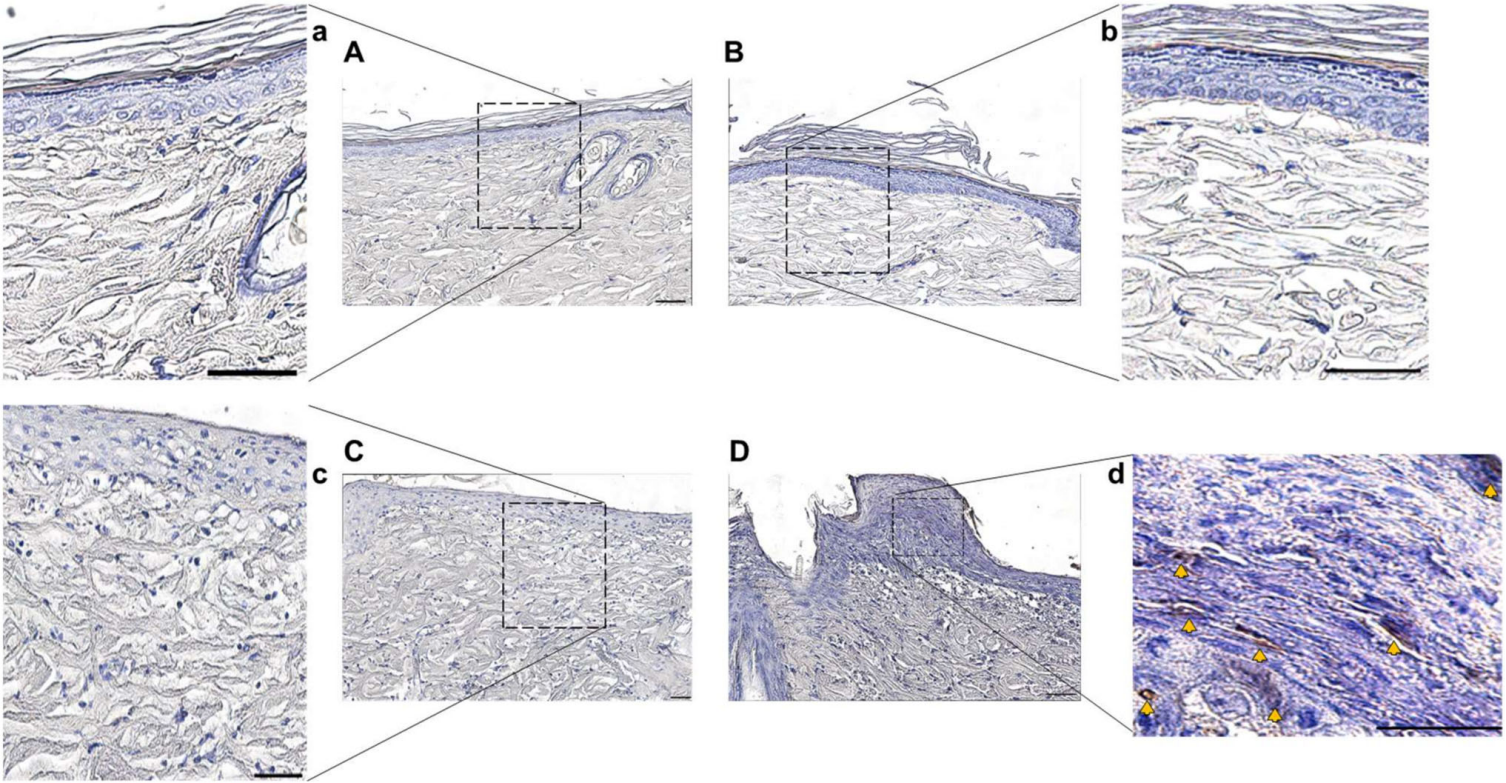
Representative images of immunohistochemistry staining for *Pseudomonas aeruginosa* (magnification: ×60). Bacterial cells are stained with brown colour. Rats in groups untreated (A), sodium lauryl sulphate (B), and cultured urine filtrate (CUF) (C) showed no expression. Bacterial cells are detected on the skin surface and inner epidermis of rats treated with CUFB (D), indicated by the yellow arrows. Scale bar for A‐D = 50 μm; (a‐d) Magnified images of boxed areas in A‐D, scale bar = 50 μm for a‐c, scale bar = 25 μm for d

Evaluation of H&E staining (Figure 3) showed normal skin histology in control rats (Figure 3A for UN and Figure 3B for SLS) with a visible stratum corneum. In contrast, the findings in skin lesions from rats treated with CUF (Figure 3C) and CUFB (Figure 3D) are irregular, with evident histological changes in the epidermis and papillary dermis. In particular, characteristic findings include the sloughing of the stratum corneum, the thickening of the viable epidermis, the increased granular layer indicating abnormal keratinocyte differentiation, the thinning of collagen fibres, and the accumulation of red blood cells in the dermis. As shown in Figure 3E, the epidermal thickness was increased in treated rats, 57.83 ± 15.99 μm for CUF and 66.32 ± 13.07 μm for CUFB, compared with UN and SLS control rats, 57.83 ± 15.99 μm and 66.32 ± 13.07 μm, respectively. However, differences were only statistically significant between rats in SLS and CUFB groups (*P* = .031). Infiltration of neutrophils and macrophages in rat skin was confirmed by immunohistochemical analysis of specific markers, namely MPO and MHC‐II, respectively. Skin samples from rats in CUF and CUFB groups showed positive staining for both MPO (Figure 4) and MHC‐II (Figure 5). In particular, there was a significant increase in the number of MHC‐II positive cells in the CUFB group compared with CUF (*P* = 0.011, Figure 5E). The staining of tissues in the CUFB group also showed the presence of bacteria on the skin surface and inner epidermis (Figure 6). No immunoreactivity to any of the markers examined was detected in samples from UN and SLS groups.

## 4 DISCUSSION

This is the first study to develop a rat model of IAD, induced by the combination of urine and bacteria and characterised by persistent redness, severe barrier disruption, partial skin loss, infiltrating inflammatory cells and infection that are hallmarks of IAD pathophysiology.^31^

While the number of studies on IAD pathophysiology has increased in recent years, IAD remains an under‐recognised condition that requires attention. In particular, IAD pathophysiology in patients with urinary incontinence remains poorly understood. Urine alone is less likely to cause IAD, but the risk increases in the presence of malodorous urine because of bacterial overgrowth.^18^ In this study, we aimed to induce IAD in an animal model using s‐urine and bacteria. Considering that urinary pH is not damaging to healthy skin as a result of its innate buffering capacity^16^ and that damaged skin characterised by elevated surface pH is associated with increased susceptibility to irritants,^21^ we compromised animal skin with SLS, simulating the chemical irritation from frequent cleansing with soap. To identify the main cause of IAD development, we examined the effects of CUF, containing only the metabolites of bacterial metabolism separately (CUF group) and in combination with bacteria (CUFB group). We found that CUF can cause mild dermatitis characterised by transient redness and barrier disruption, suggesting that the metabolite content of urine from bacteria is irritating and can damage the skin. However, after treatment with urine and bacteria (CUFB group), we were able to induce IAD. In particular, bacteria increased the severity of barrier disruption and consequently delayed its recovery, causing persistent redness, partial skin loss and increased skin inflammation, as demonstrated by the infiltration of neutrophils and macrophages. Compared with a previous rat model of IAD induced by digestive enzymes and bacteria,^13^ in this experimental model bacteria could only penetrate into the epidermis, possibly attributed to the irritating potential of proteases and lipases to cause deeper damage. However, both studies highlighted the role of bacteria in IAD development.

The nature of incontinence is a key factor in developing a preventive strategy for IAD, as it can determine the timely implementation of appropriate protocols and structured skin care regimens to protect vulnerable skin and maintain skin integrity or promote healing if IAD is already present. In our study, IAD was developed on irritated skin with increased pH, and this highlights the vital role of tissue viability nurses in maintaining healthy patient skin and ensuring optimal skin care to prevent changes in skin away from the protective acidic range, for example by using pH‐balanced cleansers or other products to keep the amino acid pool of the epidermis constant, which is primarily responsible for the buffering capacity of skin that is diminished in older adults. Our findings demonstrated that bacteria are critical for IAD development. As such, inhibiting bacterial virulence factors, for example, urease activity and ammonia generation may offer another possible preventive strategy against IAD development.^24^

In summary, we have developed a novel animal model for IAD induced by urine and bacteria. We believe that this model can serve to understand in detail the mechanisms that lead to IAD in relation to urinary incontinence, guide the development of novel interventions, and test their effectiveness in preventing IAD or in promoting the healing of such lesions.

## CONFLICT OF INTEREST

Sofoklis Koudounas and Takeo Minematsu belong to the Department of Skincare Science which receives funding from Saraya Company, however, were not involved in study design, data collection, statistical analysis, or manuscript preparation.

## Data Availability

Data openly available in a public repository that issues datasets with DOIs.
